# Cardiovascular Magnetic Resonance catheterization derived pulmonary vascular resistance and medium-term outcomes in congenital heart disease

**DOI:** 10.1186/s12968-015-0130-4

**Published:** 2015-04-14

**Authors:** Kuberan Pushparajah, Aphrodite Tzifa, Aaron Bell, James K Wong, Tarique Hussain, Israel Valverde, Hannah R Bellsham-Revell, Gerald Greil, John M Simpson, Tobias Schaeffter, Reza Razavi MD

**Affiliations:** Division of Imaging Sciences, King’s College London BHF Centre, NIHR Biomedical Research Centre at Guy’s & St Thomas’ NHS Foundation Trust, Westminster Bridge Rd, London, SE1 7EH UK; Department of Congenital Heart Disease, Evelina London Children’s Hospital, Guy’s and St Thomas’ NHS Foundation Trust, London, UK

**Keywords:** Cardiovascular magnetic resonance, Catheterization, Pulmonary vascular resistance, Interventional cardiovascular magnetic resonance, Congenital heart disease

## Abstract

**Background:**

Selection of patients with congenital heart disease for surgical septation in biventricular repair or surgical palliation in functionally single ventricles requires low pulmonary vascular resistance (PVR). Where there is uncertainty, PVR can be assessed using hybrid cardiovascular magnetic resonance (CMR) and fluoroscopic (X-Ray) guided cardiac catheterizations (XMR). CMR/XMR catheterization is a validated technique for accurate assessment of pulmonary vascular resistance. However, data concerning its application in clinical practice is lacking.

**Methods:**

PVR assessments were performed in 167 studies in 149 congenital heart disease patients by CMR/XMR catheterization. Data was collated on patient demographics, procedural data, complications and outcomes. Institutional ethics approval was obtained.

**Results:**

Median age was 3.6 years (6 days - 67 years) and weight 13.8 kg (2.3 -122 kg). One hundred and eight studies were in biventricular circulations and 59 in functionally single ventricles. Median radiation dose was 0.72 mSv. A baseline Qp:Qs ≤2.75 in biventricular circulations with left-to-right shunts predicted a PVR ≥6 WU.m^2^ with 100% sensitivity and 48% specificity. Median follow up until death or last review was 4.2 years (4 days - 11 years). Eighty-four patients had a surgical or catheter intervention based on CMR/XMR catheterization findings at a median of 94 days after the study. This included successful biventricular repair at resting PVR values ≤6 WU.m^2^ and Fontan completion at ≤4 WU.m^2^.

**Conclusion:**

PVR measured by CMR/XMR catheterization allows accurate stratification for intervention in patients with congenital heart disease in both, biventricular and univentricular circulations.

## Background

Pulmonary hypertension with elevated pulmonary vascular resistance (PVR) causes significant morbidity and mortality in children [1]. It has important implications in association with congenital heart disease where an elevated PVR precludes surgery for single ventricle palliation and septation for biventricular repair. Consequently, accurate assessment of pulmonary vascular resistance is crucial to inform clinical decision-making in congenital heart disease and where invasive studies for direct measurement of pressures are still recommended [2].

There are recognized sources of error with the Fick and thermodilution techniques in assessment of PVR particularly in the presence of intracardiac shunts, impaired function and single ventricle circulations with multiple sources of pulmonary blood flow [3-5]. Combined CMR and X-ray (XMR) guided cardiac catheterizations allow for simultaneous measurement of invasive pressures and CMR derived anatomy, function and quantification of flow [6-9]. Clinical validation studies have shown that CMR/XMR catheterization is more accurate than standard cardiac catheterization using the Fick method for the assessment of PVR in pulmonary hypertension and congenital heart disease, particularly in patients receiving 100% oxygen and inhaled nitric oxide (iNO) [3,8,10].

We present results of PVR assessment by CMR/XMR catheterization, which includes analysis of indications, results and outcomes. We hypothesize that accurate PVR measurements in this way can be reliably applied in clinical decision making for septation in biventricular repair or surgical palliation in patients with functionally single ventricle physiology.

## Methods

### Patients

One hundred and forty nine pediatric and adult patients with congenital heart disease and clinical suspicion of raised PVR on clinical assessment or echocardiography underwent 167 CMR/XMR cardiac catheterizations in our institution between February 2002 and February 2012. Ethical approval was obtained by St. Thomas' Hospital Research Ethics Committee/South East London Research Ethics Committee prior to commencement of the CMR/XMR cardiac catheterization program. This study includes 16 patients from previously published data describing the early initial clinical experience of this technique [8] and 24 patients from the validation study of PVR calculation using CMR/XMR [3].

### Technique

The techniques of CMR/XMR catheterization have been previously described [3,8,10-12]. In our hybrid laboratory we used a 1.5 T MR-scanner (Achieva, Philips, Best, Netherlands) and a Philips BV Pulsera cardiac X-Ray unit. All procedures were performed under general anesthesia with sevofluorane and intravenous remifentanyl. A heparin bolus of 50 IU/Kg was given with activated clotting time (ACT) monitoring once vascular access was obtained. Patients underwent either solely CMR guided catheterization or XMR catheterization.

For XMR catheterization procedures, catheters were positioned in the appropriate vessels and chambers using guide-wires where necessary under X-Ray guidance. Once right heart catheterization was completed, CMR compatible catheters (Wedge catheter, Arrow, Reading, PA, USA) were left in place in the pulmonary artery for continuous hemodynamic pressure monitoring and the patient was transferred to the CMR scanner on the sliding table.

For CMR guided catheterization, following an initial reference scan, an interactive sequence was used for determining and saving reference planes for catheter guidance. During passive catheter tracking, a 2D steady state free precession (SSFP) sequence (Balanced- FFE, TE 1.45 ms, TR 2.9 ms, matrix 128×128), with a temporal resolution of 10–14 frames per second was used in which the catheter tip is seen on filling the angiographic balloon with 1 ml of carbon dioxide [8,9]. For these patients, catheter placement for right heart catheterization was performed solely under CMR guidance.

To obtain flow measurements in major vessels, through plane velocities were measured by means of phase contrast gradient echo sequences perpendicular to the long axis planes of the vessel with either breath-hold or free-breathing flow-sensitive segmented k-space fast field echo sequence (approximate echo time 3 ms, approximate repetition time 5 ms, matrix 128×256, field of view 250–350 mm, flip angle 15°, number of signal averages 3, retrospective gating, 40 acquired phases).

### Pulmonary vascular resistance (PVR)

Invasive pressure and phase contrast flow were measured simultaneously and repeated under different physiological conditions. Throughout, we aimed for hemodynamic stability in the patient and normocarbia (PaCO_2_ 4-5kPa). Baseline measurements were made in 30% inspired oxygen. Patients undergoing reversibility studies had repeat measurements 20 minutes after administration of inhaled nitric oxide (iNO) at 20 ppm and 100% inspired oxygen. Pressures where measured with breatholds and flows were measured consistently as either free-breathing or with breatholds. Where breatholds were used, they were limited to no longer than 15 seconds and normocarbia was maintained as observed using the measured end-tidal CO2.

Pulmonary vascular resistance was calculated as below:

$$ \mathrm{P}\mathrm{V}\mathrm{R}\left(\mathrm{W}\mathrm{U}.{m}^2\right)=\frac{\left[\mathrm{mean}PA\mathrm{pressure}\left(\mathrm{mmHg}\right)-\mathrm{mean}LA\mathrm{pressure}\left(\mathrm{mmHg}\right)\right]}{\mathrm{indexed}PA\mathrm{flow}\left(\mathrm{L}/ \min /{\mathrm{m}}^2\right)} $$

Left atrial (LA) pressure was either measured directly or obtained from a pulmonary capillary wedge pressure (PCWP) accepting that PCWP only remains a reliable measure of LA pressure at values <15 mmHg. Units were expressed as Wood units (WU) indexed to body surface area (WU.m^2^). Mean pulmonary artery (PA) pressure was either measured in the main PA or branch PAs. Where branch PA flows and pressures were used, we measured individual lung PVR and calculated a total PVR.

$$ \frac{1}{R_{Total}}=\frac{1}{R_{RPA}}+\frac{1}{R_{LPA}} $$

### Statistical analysis

Statistical analysis was performed using SPSS software (version 18; Chicago, Ill). Normally distributed data are reported as mean ± standard deviation and data with skewed variables as median and range. Statistical differences were assessed by paired t-test analysis. The receiver operator characteristic (ROC) curve of the baseline Qp:QS and PVR at baseline was performed. Statistical significance was set a priori at p <0.05. 

## Results

### Patients

Median age was 3.6 years (6 days-67 years) and weight 13.8 kg (2.3 -122 kg). There was a wide range of underlying diagnoses described in Table 1. Seventeen patients had an associated diagnosis of trisomy 21. Ninety-seven patients had previous surgical or catheter interventions. Fifteen had a single repeat CMR/XMR study, and 3 patients had 2 repeat CMR/XMR studies. One hundred and fifty four were combined XMR catheterizations and 13 were solely CMR guided cardiac catheterizations. Median radiation dose for CMR/XMR catheterization was 3.6 Gycm^2^ (0.72 mSv) (range 0–57.5 Gycm^2^), with a median screening time of 11.2 minutes (range 0–58.2 minutes). There were 2 procedural complications; pulmonary hemorrhage and pulmonary embolism with good recovery and no procedural deaths.

**Table 1:** **Primary structural cardiac lesions of the study population**

**Biventricular**	**N = 99**	**Univentricular**	**N = 50**
L-R shunts		Unbalanced AVSD	6
ASD	4	Hypoplastic left heart syndrome	20
VSD	27	complex	1
Partial AVSD	2	Tricuspid atresia	6
Complete AVSD (includes associated coarctation/arch hypoplasia, PS)	22	DORV (univentricular)	8
PDA	1	PA/IVS	4
PAPVR	1	DILV	3
Supracardiac TAPVD	1	ccTGA (univentricular)	2
AP window	1		
DORV	3		
Right heart obstructive		Complex (Biventricular)	
Pulmonary stenosis	4	Truncus arteriosus	1
Branch pulmonary stenosis	9	TGA/VSD/Coarctation	1
Tetralogy of Fallot	7	Other	
Pulmonary atresia/intact ventricular septum	1	Cortriatrium	1
Absent PV	1	Scimitar syndrome	7
Left heart obstructive lesions		Ebstein’s anomaly	1
Aortic arch hypoplasia	1	Pentalogy of Cantrell	1
		ccTGA	2
	85		12

Four patients were lost to long term follow up having moved abroad. Follow up until death or last medical review was available for the remaining patients over a median of 4.2 years (4 days to 11 years). The 4-day duration was related to a death from extracardiac complaints explained later in the manuscript. Eighty-four studies led to a cardiac intervention (catheter or surgical) at a median interval of 94 days (0–414 days). The remaining patients either had medical or conservative treatment.

### Pulmonary vascular resistance and clinical outcomes

Pulmonary vascular resistance results are reported separately for biventricular and functionally single ventricle circulations.

### Biventricular circulations

**Figure 1:**
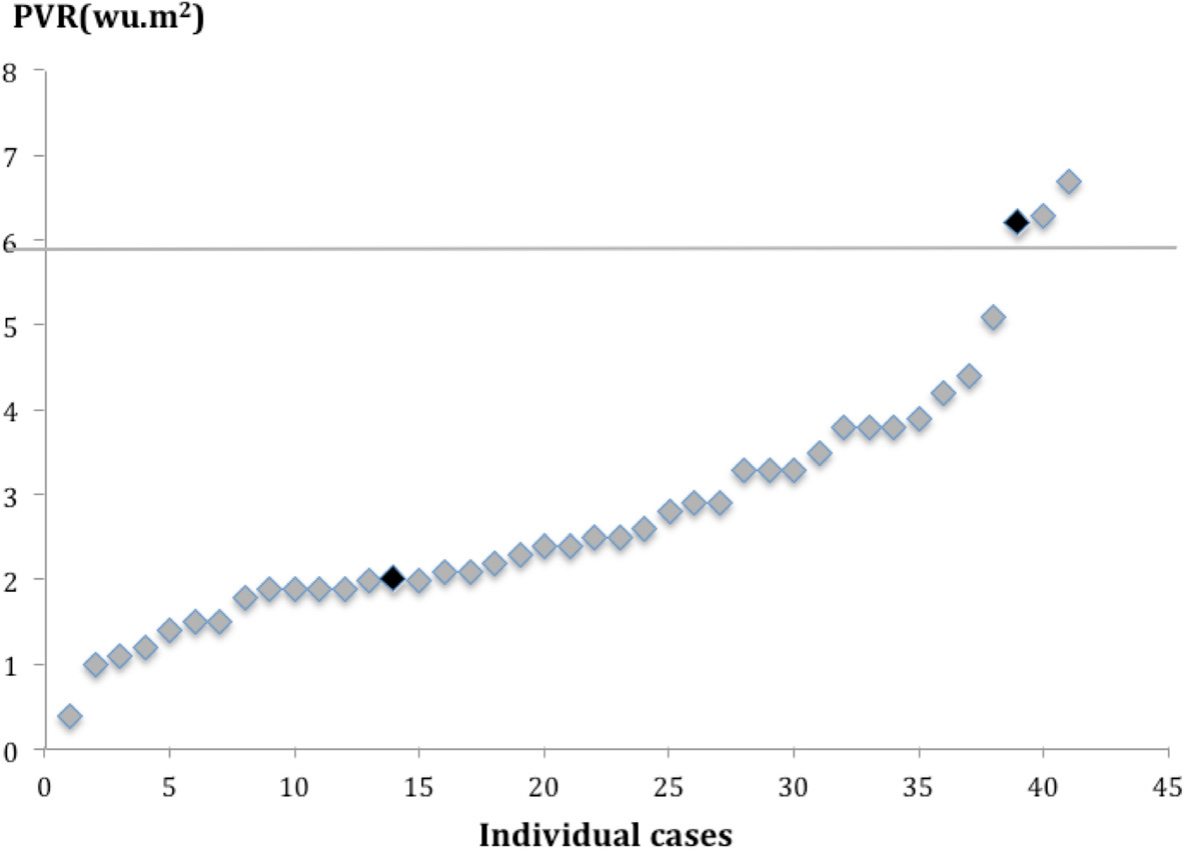
**PVR in patients with biventricular circulations and left to right shunts undergoing interventions post CMR/XMR catheterization.** Patient death is marked in black.

**Figure 2:**
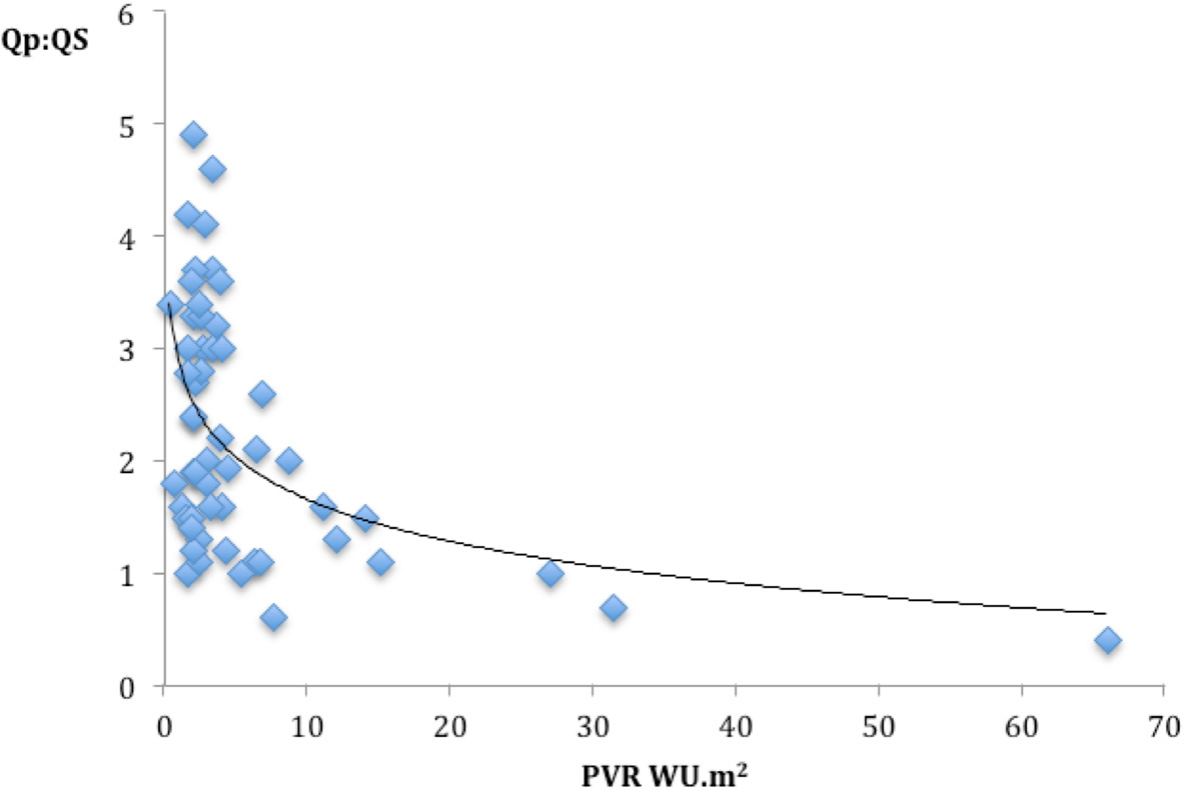
**Relationship of PVR to Qp:Qs in biventricular circulations with aorta-to-pulmonary shunts.**

**Figure 3:**
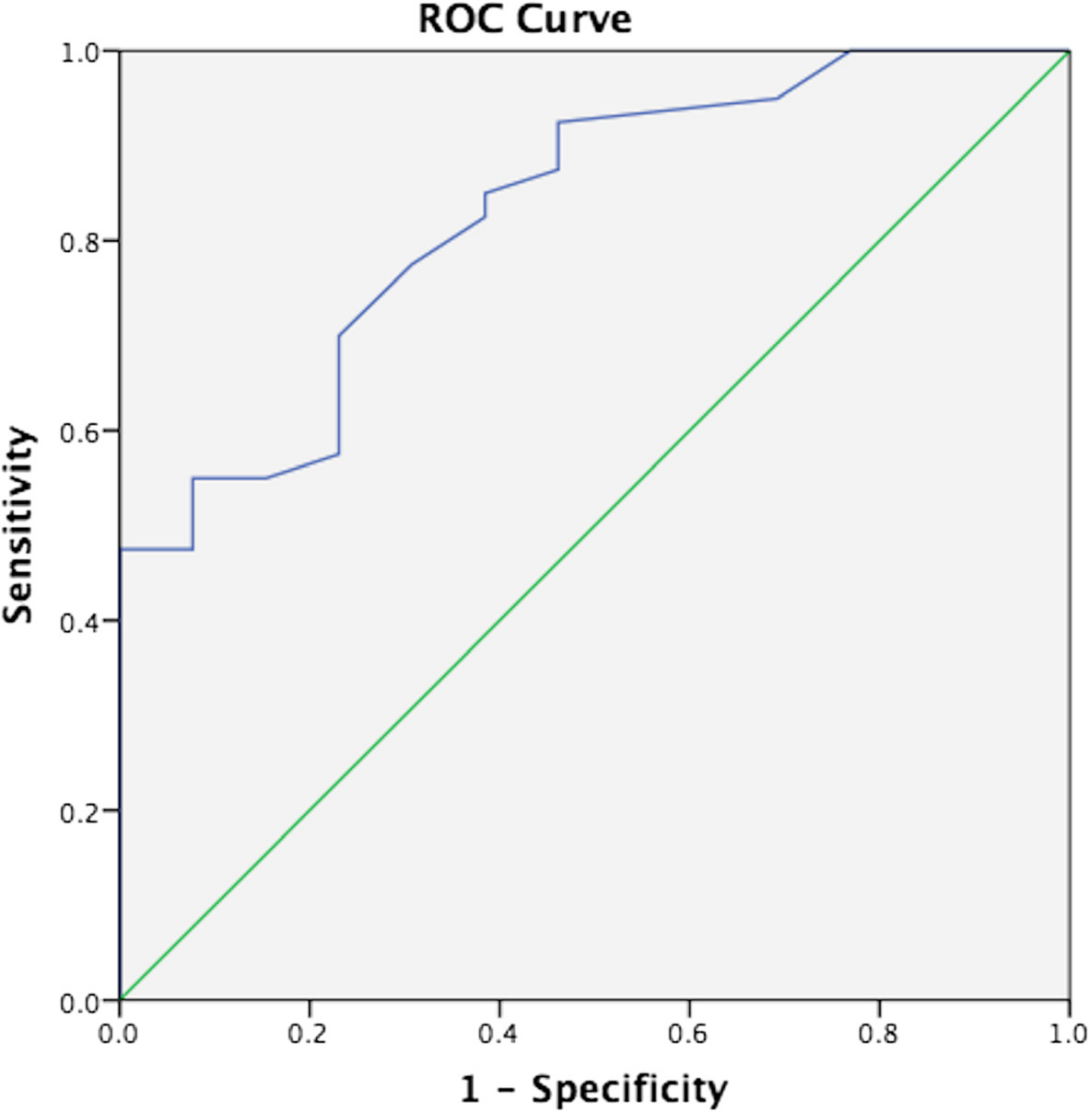
**ROC curve for the relationship of PVR to Qp:Qs in biventricular circulations with aorta-to-pulmonary shunts.** The area under the curve is 0.829.

One hundred and eight PVR studies were performed in biventricular circulations. Forty-seven led to a cardiac intervention and 61 followed a conservative path or non-cardiac intervention. Median PVR was 2.8 WU.m^2^ (range 0.4-66.0). Sixty-seven studies were in a group with lesions that allowed shunting between the systemic and pulmonary circulations or potentially vice-versa. Of these, 41 went on to have a cardiac catheter or surgical intervention to eliminate or substantially reduce the shunt. The PVR values of this interventional group of patients are displayed in Figure 1. ROC analysis showed that a baseline Qp:Qs ≤2.75 in biventricular circulations with left-to-right shunts predicted a PVR ≥6 WU.m^2^ with 100% sensitivity and 48% specificity (Figure 2 and Figure 3). This translates to a negative predictive value of 100% and a positive predictive value of 37%.

Reversibility with 20 ppm iNO and 100% oxygen were performed in 52 studies with systemic to pulmonary shunts, with a fall in mean PVR from 3.3 to 2.9 WU.m^2^(p = 0.03) and a rise in mean Qp:Qs ratio from 2.2 to 2.4 (p = 0.02). Further interrogation showed a mean reduction of PVR by 12.7% (range 0-38%) in those undergoing interventions and 10.8%(range 0-34%) in those who were managed medically (p = 0.4).

A baseline PVR of around 6 WU.m^2^ and lower was considered suitable for biventricular surgical repair, accepting the need for fenestrated closure of left-to-right (L-R) shunt lesions in the borderline cases. Those with a PVR < 6 WU.m^2^ remain alive post intervention with only one death, unrelated to PVR (PVR of 2 WU.m^2^) in a patient with pulmonary atresia, ventricular septal defect and major aortopulmonary collaterals who required multiple surgical and catheter interventions to address residual proximal branch PA narrowing. This low PVR group included four adult patients with chronic L-R shunts (AVSDs or VSD) who went on to have surgery at a mean age of 46 years (range 35.6-52.8 yrs) and remain alive with no evidence of pulmonary hypertension.

Six patients with biventricular circulations had a fenestrated, rather than complete closure of their intracardiac shunt lesions due to the marginally elevated PVR, three of whom had a PVR just above 6 WU.m^2^ (6.2, 6.3 and 6.7 WU.m^2^). Of these, one patient died following surgical intervention despite fenestrated closure, from persistent pulmonary hypertension. This patient had an elevated pre-surgical PVR of 6.2 WU.m^2^ falling to 4.8 WU.m^2^ in response to maximum pulmonary vasodilation during the PVR study and a Qp:Qs that remained static at 1.1.

Of the 26 patients with systemic to pulmonary shunt lesions who were not operated, 11 were because of an elevated PVR of >6 WU.m^2^ (median 15 WU.m^2^ range 6.6-66 WU.m^2^) and remain alive on medical treatment. The remaining 15 patients with PVR <6 WU.m^2^ (median 2.4 WU.m^2^, range 0.6-5.3 WU.m^2^) were managed conservatively as the defects were judged to be small. There was one death in this low PVR group in a patient with a ventricular septal defect, VACTERL association (**V**ertebral anomalies, **A**nal atresia, **C**ardiac defects, **T**racheo-oesophageal fistula and or **E**osophageal atresia, **R**enal and radial anomalies and **L**imb defects) and chronic lung disease (PVR 1.6 WU.m^2^) with a laryngeal cleft and airway difficulties.

Forty one patients had a biventricular circulation without a lesion that allowed shunting between the systemic and pulmonary circulations or potentially vice-versa (median PVR 3.1 WU.m^2^ (0.6-31.4 WU.m^2^). Four patients with low PVR went on to have catheter or surgical intervention with 3 stenting of the right ventricular outflow tract or branch pulmonary arteries, and one balloon of the pulmonary valve. Two patients with a PVR greater than 6 WU.m^2^ (19 and 6.2 WU.m^2^) underwent catheter-guided occlusion of aortopulmonary collaterals. The patient with a PVR of 19 WU.m^2^ had a previously repaired hypoplastic aortic arch and died from pulmonary hypertension despite embolization of a right MAPCA and initiation of sildenafil. The second patient with Scimitar syndrome survived.

Of the remaining patients without shunt lesions, eight had no cardiac interventions but underwent a liver transplant with good outcomes. The remaining 27 patients have had no interventions and remain alive at last follow up. Eleven had normal PVR and were managed conservatively (median PVR 1.8 WU.m^2^, range 1.0-3.4) and 16 were treated with oral pulmonary vasodilators for varying periods of time (median PVR 8.0, range 2.0-42.3 WU.m^2^).

### Functionally univentricular circulations

Standard care for the preoperative assessment of patients undergoing single ventricle surgical palliation at our center has not involved cardiac catheterization since 2002. Patients progressing along the path of surgical single ventricle palliation undergo a CMR with simultaneous measurement of central venous pressure by means of jugular venous cannulation. Fifty-nine catheter studies were performed in 50 patients with functionally univentricular circulations where there were concerns regarding an elevated PVR. Thirty-seven studies led to a cardiac intervention, and 22 followed a conservative course. Median PVR was 2.3 WU.m^2^ (range 0.9-6.6 WU.m^2^).

**Figure 4:**
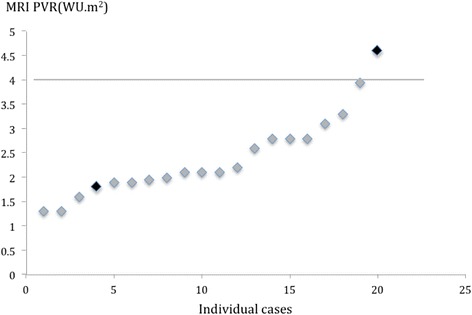
**PVR in patients with univentricular circulations undergoing staged surgical palliation to completion of Fontan post CMR/XMR catheterization.** Patient deaths are marked in black.

Forty-two studies were performed in 35 patients prior to completion of Hemifontan or Fontan. Twenty patients went on to have completion of the Fontan circulation (Figure 4) despite a raised PVR in 4 patients (PVR 4.6, 3.9, 3.3 and 3.1 WU.m^2^). The median pulmonary artery pressure pre-Fontan was 10.4 mmHg (range 6-18 mmHg). It is our institutional practice to routinely fenestrate all Fontans. There was one long-term death in a patient with a PVR of 4.6 WU.m^2^, reducing to 3.6 WU.m^2^ with iNO and 100% oxygen undergoing a high risk Fontan on sildenafil therapy. There was one death (PVR 1.8 WU.m^2^) due to plastic bronchitis 85 days post completion of Fontan.

Of the 15 patients who did not have Fontan completion, 3 had a PVR > 4 WU.m^2^ and were medically treated for pulmonary hypertension, with one death. Of patients managed conservatively with a PVR < 4 WU.m^2^, one is awaiting surgery for completion of Fontan upon reduction of body mass index, and two were deemed unsuitable for Fontan completion despite a PVR <4 WU.m^2^ due to primary ciliary dyskinesia/cystic fibrosis in one, and branch pulmonary arteries that were considered too small in the other. Six patients remain palliated with cavopulmonary shunts and have not been referred for pre-Fontan assessment as yet. Three patients died pre Fontan from complications unrelated to PVR. The first patient died following surgery for unrelated endocarditis of the mitral valve. The second patient had a sudden arrhythmic death. The third patient was a neonatal death following withdrawal of care 40 days after a redo of pulmonary artery banding and atrial septectomy due to multiple congenital anomalies and associated complications from necrotising enterocolitis.

Fifteen patients had 17 studies post-Fontan completion (median 2.1 WU.m^2^, range 0.9-4.3 WU.m^2^) of whom 12 were treated conservatively or with medical therapy. The remaining 3 led to catheter interventions, which consisted of coil occlusion of aortopulmonary collaterals (PVR 2.2 WU.m^2^), lateral tunnel fenestration creation and stenting (PVR 2.9 WU.m^2^) and fenestration closure (PVR 1.4 WU.m^2^). All remain alive.

## Discussion

In this study, we report CMR/XMR derived PVR values and long-term outcomes over a median of 4.2 years in a large cohort of patients with biventricular and single ventricle physiology. This provides a basis for accurate stratification of patients with congenital heart disease being considered for interventions.

### Pulmonary vascular resistance

Our data demonstrates that patients with significant L-R shunt lesions with PVR values of up to 6 WU.m^2^ were shown to have good long-term outcomes following surgical repair even if this was carried out well into adulthood. There was however a need for fenestrated closure in those with moderately raised PVR. The commonly accepted criteria for intervention in patients with moderately raised PVR is based on consensus opinion [13] and suggests that patients with intracardiac shunts are suitable for surgical biventricular repair if the resting PVR is less than 6 WU.m^2^. However, long-term outcome data using accurate PVR assessment techniques in congenital heart disease are lacking. Careful selection of patients is important as closure of atrial septal defects in patients with elevated pulmonary artery pressures without PVR assessment has resulted in poor outcomes [14].

As the thresholds for interventions are stretched, it is important to remember that pulmonary hypertension occurring in the postoperative patient with congenital heart disease carries a 23% mortality which is far worse [15] than in unoperated patients who develop Eisenmenger syndrome. Therefore, careful selection of appropriate patients is crucial to avoid prematurely lowering life expectancy. Our data provides objective support for the use of the current threshold for intervention of 6 WU.m^2^. One out of 3 patients with a PVR > 6 WU.m^2^ died from pulmonary hypertension having had an intervention in comparison to the 11 patients in the same PVR group who all remain alive without any medical or catheter intervention. Careful selection of patients using detailed PVR assessment has allowed us to successfully perform late repair of VSDs in adults who may have otherwise been deemed too high risk. This is in keeping with previous reports [16].

Defining PVR thresholds is also vital for patients being put forward for single ventricle surgical palliation where a lower PVR is crucial for adequate pulmonary blood flow in the absence of a subpulmonary ventricle. In our experience, single ventricle palliation was shown to result in favorable outcomes in patients with a PVR of less than 4 WU.m^2^. The one patient in our cohort with a PVR >4 WU.m^2^ who underwent single ventricle palliation did not survive. This is in keeping with the original ‘10 commandments’ as described by Choussat which include a PVR <4 WU.m^2^ and mean pulmonary artery pressures <15 mmHg. Many of these commandments have been dispensed with over the years, but Hosein et al. [17] have demonstrated increased adverse outcomes post Fontan in the presence of pre surgical elevated mean pulmonary artery pressures >16 mmHg and impaired systolic ventricular function. Hosein et al. however noted the difficulty in assessing PVR accurately in these patients and were not able to correlate this to outcomes, both of which we have been able to achieve with CMR/XMR guided catheterization even were there were multiple sources of pulmonary blood flow. We have also demonstrated good outcomes following Fontan completion with mean PA pressures of up to 18 mmHg where the measured PVR was in fact low, though caution must be exercised in interpretation of these data given the small number of patients.

### Pulmonary vascular resistance reversibility

There is no fixed guideline for the degree of reversibility in PVR in response to pulmonary vasodilators as a threshold for a positive response in pediatric patients with congenital heart disease. However, consensus suggests a reduction in PVR of 20% as a positive response [13]. We found that no significant differences in the degree of reduction of PVR in response to pulmonary vasodilators in those undergoing interventions compared to those managed medically. In our population, the underlying value of the baseline PVR was probably more meaningful than response to pulmonary vasodilators in terms of the decision to intervene with good long-term outcomes.

### Non-invasive assessment of PVR

Patients with unrestricted left to right shunts can have PVR assessed non-invasively as we have previously outlined [18] in a study of 26 of these patients. We previously observed that a Qp:Qs of ≤2.5:1 predicted a PVR of ≥3.5 WU.m2 with sensitivity 100%, and specificity 83%. We have expanded this to include more datasets and refined this value such that a baseline Qp:Qs ≤2.75 in biventricular circulations predicted a PVR ≥ 6 WU.m2 with 100% sensitivity and 48% specificity. Therefore in biventricular circulation with left to right shunts, it would be possible to avoid cardiac catheterization if the Qp:Qs can be measured accurately and is greater than 2.75. However a lower Qp:Qs is not sufficiently predictive of a high PVR and these patient should still undergo assessment by cardiac catheterization. Importantly, a Qp:Qs ≤2.75 in a patient with a small, restrictive PDA, VSD, or ASD is not necessarily indicative of elevated pulmonary vascular resistance. For instance, a high Doppler peak velocity in a small PDA or VSD in conjunction with normal systolic interventricular septal configuration on two-dimensional echocardiography provides presumptive evidence of normal pulmonary vascular resistance.

### Radiation exposure

Solely CMR guided catheterization is possible [6,8,10] but is limited in congenital heart disease where catheter manipulation is often performed with the aid of non-CMR compatible guidewires under X-ray guidance due to the complex anatomy. The variation in our practice for using solely guided CMR compared to XMR was related in part to anatomical constraints and the experience of the operator. Despite the need for X-ray fluoroscopy, the radiation doses in CMR/XMR catheterization remain low. The median radiation exposure of 0.72 mSv compares favorably against conventional fluoroscopic diagnostic catheterization in contemporary literature of 10.8 mSv [19]. The role for solely CMR guided catheterisation will expand in line with developments in CMR compatible catheters and guide wires where there are developments from a number of companies with CE marking achieved or imminent.

### Applicability

The need for accurate assessment of PVR is more pressing given the complexity of patients undergoing treatment due to improved surgical and interventional outcomes for congenital heart disease. CMR/XMR catheterization is a useful clinical tool for accurate PVR assessment, but requires specialist hardware and a skilled multidisciplinary team. Where there are cost implications for hybrid XMR suites, XMR catheterization could still be performed as a single procedure in separate laboratories provided there is close proximity between the catheter laboratory and CMR scanner and that patient safety is not compromised. Finally, performing cardiac catheterisation in patients with elevated PVR can be challenging and there are reports of occasional mortality. Therefore, appropriate pre-procedure assessment with skilled anaesthetic support must take place.

### Limitations

All patients referred for CMR/XMR evaluation had a high suspicion of abnormal hemodynamics requiring more thorough evaluation and therefore represent a selected population. It should be stressed that it is our institutional practice to base surgical decision-making for these patients on clinical findings, echocardiography and CMR data without routine diagnostic catheterization [7,8,12,20,21] in keeping with the contemporary congenital heart practice of others. This highlights the targeted role of CMR/XMR evaluation of PVR.

It is our institutional practice to perform all cardiac catheter procedures under general anesthesia with 30% fraction of inspired oxygen, which may affect PVR. Also due to potential effects of positive pressure ventilation in single ventricle patients we are careful to eliminate positive end expiratory pressure particularly in this group when pressure measurements are being done.

## Conclusion

CMR/XMR catheterization is safe and accurate tool in the measurement of PVR and allows risk stratification of patients with congenital heart disease being considered for interventions. We have identified discrete PVR thresholds below which good long-term outcome can be achieved following surgical repair or palliation.

## Abbreviations

HLHS: Hypoplastic left heart syndrome
CMR: Cardiovascular magnetic resonance
XMR: Combined X-ray and CMR
PVR: Pulmonary vascular resistance
LA: Left atrium
PCW: Pulmonary capillary wedge
iNO: inhaled nitric oxide
VSD: Ventricular septal defect
AVSD: Atrioventricular septal defect
MAPCA: Major aortopulmonary collateral
